# Supplementary material for: An operating principle of the cerebral cortex, and a cellular mechanism for attentional trial-and-error pattern learning and useful classification extraction
Source: Front Neural Circuits. 2024 Mar 5;18:1280604. doi: 10.3389/fncir.2024.1280604 (PMC10950307; doi:10.3389/fncir.2024.1280604)
Supplement: Supplementary file 1 [file Data_Sheet_1.docx]

Supplementary Material

An operating principle of the cerebral cortex, and a cellular mechanism for attentional trial-and-error pattern learning and useful classification extraction

Marat M. Rvachev*

*** Correspondence:** rvachev@alum.mit.edu

# Supplementary Data

The following Perl script generates data for Figures 10C and 10D, by repeatedly executing the Perl program named “Section5Fig10CD.pl”, listed below.

# Perl script to produce data for the charts in Figures 10C and 10D

$N_i = 100; # The number of external inputs

$N_o = 10; # The number of output minicolumns

$N_p = 100; # The number of simulated patterns per simulation

$numSimulations = 50; # The number of simulations to run and average over for Figure 10C

$output = "Figure10C.txt"; # Output file name for Figure 10C results

foreach $n_c (4){ # Synaptic cluster size

foreach $N_e (4..6){ # The number of objects in an input pattern

foreach $F (0..16){ # F, the scaling factor for L2/3 to L5b connectivity

print `perl Section5Fig10CD.pl $numSimulations 3 $N_i $N_o $N_p $n_c $N_e $F 1 $output`;

}

}

}

$numSimulations = 10; # The number of simulations to run and average over for Figure 10D

$output = "Figure10D.txt"; # Output file name for Figure 10D results

foreach $n_c (4..6){ # Synaptic cluster size

foreach $N_e (1..8){ # The number of objects in an input pattern

print `perl Section5Fig10CD.pl $numSimulations 1 $N_i $N_o $N_p $n_c $N_e 1 1 $output`;

}

}

The Perl program “Section5Fig10CD.pl” below should be placed into a file “Section5Fig10CD.pl”, and is executed by the script above.

# System configuration for the results in the paper:

# Operating system: Microsoft Windows 10 Pro, version 10.0.19045

# Perl: v5.32.1, built for MSWin32-x64-multi-thread (Strawberry Perl distribution)

#

# Command line arguments: $numSimulations -- the number of simulations to run and average over, $H -- parameter

# specifying configuration (3 for Figure 10C, 1 for Figure 10D), $N_i -- the number of external inputs,

# $N_o -- the number of minicolumns, $N_p -- the number of simulated patterns per simulation,

# $n_c -- the number of synapses in a synaptic cluster, $N_e -- the number of objects ("blobs")

# in an input pattern, $F -- scaling factor for L2/3 to L5b connectivity (see "Materials and Methods"),

# $corrPatterns -- a flag to toggle correlation between the "XXXXX" and "XXOXX" patterns for Figure 10C

# (1 for the results in the paper), $outputFile -- the output file name for the results.

if ($ARGV[0] > 0){ ($numSimulations, $H, $N_i, $N_o, $N_p, $n_c, $N_e, $F, $corrPatterns, $outputFile) = @ARGV; }

my @layer2, @inpPattern;

if($H == 1){ # $H = 1 is the Figure 10D configuration

$internalWorlds = 2;

$layer2[0] = "11111"; # The object in a pattern

$inpPattern[0] = $layer2[0]; # copy of the above

}else{ # $H = 3 is the Figure 10C configuration (here $H is the number of L2/3 neurons per minicolumn + 1)

$internalWorlds = 1;

$layer2[0] = "11111"; # Recognizable object for the first L2/3 neuron, an object in a pattern

$layer2[1] = "11011"; # Recognizable object for the second L2/3 neuron, an object in a pattern

$inpPattern[0] = $layer2[0]; # Copies of the above

$inpPattern[1] = $layer2[1];

}

$minBlob = length($inpPattern[0]); # The size of an input object

$N_s_max = 20000; # The number of basal synapses per output neuron

$maxClustersPerNeuron = $N_s_max / $n_c; # The number of clusters per output neuron

if ($N_e == 1 && $N_p >= $N_i - $minBlob + 1){

$N_p = $N_i - $minBlob + 1; # E.g.: for $N_i = 100, $minBlob = 5, and $N_e = 1, can't generate more than 96 different patterns

}

srand(123456789); # Set the random number seed

for($iSimulation = 0; $iSimulation < $numSimulations; $iSimulation++){ # Loop over simulations

%clusters = {}; %clustersCtr = {}, %clustersWt = {}; %subClusters = {};

@patterns = (); @patternIndexes = (); @correctOutputNeuron = ();

generateClusters(); # Generate clusters

generatePatterns(); # Generate patterns

# Training

for(my $iTrainingPattern = 0; $iTrainingPattern < $N_p ; $iTrainingPattern++ ) { # Loop over $N_p training patterns

# Generate a trial firing

my @firingOutputNeurons = (0) x ($N_o * $internalWorlds);

my $iMotorNeuronFiring = $correctOutputNeuron[$iTrainingPattern]; # The minicolumn firing during training

$firingOutputNeurons[$iMotorNeuronFiring] = 1;

if($internalWorlds == 2){

$firingOutputNeurons[$iMotorNeuronFiring + $N_o] = 1;

}

$flagTrialFiring = 1;

$reward = 1;

my @currentTrainingPattern = @{ $patterns[$iTrainingPattern] };

my $stringTrainingPattern = join ("", @currentTrainingPattern);

my @addedResponse = ();

for (my $n = 0; $n < $H-1; $n++) { # Generate L2/3 output as L5b input for Figure 10C

push (@addedResponse, layer2response($stringTrainingPattern, $n));

}

push (@currentTrainingPattern, @addedResponse);

my @currentTrainingPatternIndexes = @{ getFiringInputsOrOutputsIndexes(\@currentTrainingPattern) };

updateClusters ($reward, \@currentTrainingPatternIndexes, \@firingOutputNeurons); # Update cluster weights

}

for(my $iTestingPattern = 0; $iTestingPattern < $N_p ; $iTestingPattern++ ) { # Loop over testing patterns

my @currentTestingPattern = @{ $patterns[$iTestingPattern] };

my $stringTrainingPattern = join ("", @currentTestingPattern);

my @addedResponse = ();

for (my $n = 0; $n < $H-1; $n++) { # Generate L2/3 output as L5b input for Figure 10C

push (@addedResponse, layer2response($stringTrainingPattern, $n));

}

push (@currentTestingPattern, @addedResponse);

my @currentTestingPatternIndexes = @{ getFiringInputsOrOutputsIndexes(\@currentTestingPattern) };

my @firingOutputWeights = @{getFiringOutputWeights (\@currentTestingPatternIndexes)};

my $firingOutputNeuron, $firingOutputNeuron2;

if($internalWorlds == 1){

$firingOutputNeuron = getMaxWeightNeuron (\@firingOutputWeights);

}else{

my @firingOutputWeights1 = @firingOutputWeights[0 .. ($N_o - 1)];

$firingOutputNeuron = getMaxWeightNeuron (\@firingOutputWeights1);

my @firingOutputWeights2 = @firingOutputWeights[$N_o .. (2 * $N_o - 1)];

$firingOutputNeuron2 = getMaxWeightNeuron (\@firingOutputWeights2);

}

if ( $firingOutputNeuron == $correctOutputNeuron[$iTestingPattern] && ($internalWorlds == 1 ||

($internalWorlds == 2 && $firingOutputNeuron2 == $correctOutputNeuron[$iTestingPattern] ) )){

$sumCorrectClass++;

}else{

$sumIncorrectClass++;

}

$totalTests++;

$totWtCorr += $firingOutputWeights[ $correctOutputNeuron[$iTestingPattern] ]; # Keep track of output strength

# for correct and incorrect outputs

foreach my $wgt ( @firingOutputWeights ) { $totWtIncorr += $wgt; }

$totWtIncorr -= $firingOutputWeights[ $correctOutputNeuron[$iTestingPattern] ];

}

$cntSimulation++;

}

$percCor = sprintf ("%.3f", $sumCorrectClass/$totalTests*100);

$percIncor = sprintf ("%.3f", $sumIncorrectClass/$totalTests*100);

$corrClassWt = sprintf ("%.3f", $totWtCorr / $totalTests);

$incorrClassWt = sprintf ("%.3f", $totWtIncorr / $totalTests / ($N_o - 1) );

if ( $incorrClassWt > 0) {$corrToIncorrWtRatio = sprintf ("%.2f", $corrClassWt / $incorrClassWt);}

$out = "numSim = $cntSimulation, H = $H, N_i = $N_i, N_o = $N_o, N_p = $N_p, n_c = $n_c, N_c(created) = ".

"$globalClusterCounterPerNeuron, N_e = $N_e, F = $F, corrPatterns = $corrPatterns;

\% correct: $percCor, \% incorrect: $percIncor,

avg output weight for correct class: $corrClassWt, avg output weight for incorrect class: $incorrClassWt, " .

"correct to incorrect output weight ratio = $corrToIncorrWtRatio

";

print "$out";

$out =~ s/([^0-9\.]+)/\ /msg; # Remove non-numbers from output to produce a brief output version

print "Numbers output:

$out\n";

open (FILE, ">>$outputFile"); # Append abbreviated output version to the output file

print FILE "$out\n";

close (FILE);

exit;

sub generateClusters(){ # Generate clusters on the neurons

for(my $iOutputNeuron = 0; $iOutputNeuron < $N_o * $internalWorlds; $iOutputNeuron++) { # Loop over output neurons

my $i1, $i2, $i3, $i4, $i5, $i6, $i7, $createdClusterCtr; # This program supports the synaptic cluster size up to 7

$i1 = $i2 = $i3 = $i4 = $i5 = $i6 = $i7 = $createdClusterCtr = 0;

for(my $iCluster = 0; $createdClusterCtr < $maxClustersPerNeuron; $iCluster++ ) {

LABEL7:

$i1 = randNH($N_i, $H);

$i2 = randNH($N_i, $H);

$i3 = randNH($N_i, $H);

$i4 = randNH($N_i, $H);

$i5 = randNH($N_i, $H);

$i6 = randNH($N_i, $H);

$i7 = randNH($N_i, $H);

if ($n_c <= 6){$i7 = 9999;} # Set unused input indexes to 9999

if ($n_c <= 5){$i6 = 9999;}

if ($n_c <= 4){$i5 = 9999;}

if ($n_c <= 3){$i4 = 9999;}

if ($n_c <= 2){$i3 = 9999;}

if ($n_c <= 1){$i2 = 9999;}

if ($n_c >= 2) { if ( $i1 == $i2 ){ goto LABEL7; } } # Regenerate cluster if it has duplicate inputs

if ($n_c >= 3) { if ( ($i1 == $i3) || ($i2 == $i3) ){ goto LABEL7; } }

if ($n_c >= 4) { if ( ($i1 == $i4) || ($i2 == $i4) || ($i3 == $i4) ){ goto LABEL7; } }

if ($n_c >= 5) { if ( ($i1 == $i5) || ($i2 == $i5) || ($i3 == $i5) || ($i4 == $i5) ){ goto LABEL7; } }

if ($n_c >= 6) { if ( ($i1 == $i6) || ($i2 == $i6) || ($i3 == $i6) || ($i4 == $i6) || ($i5 == $i6) ){ goto LABEL7; } }

if ($n_c >= 7) { if ( ($i1 == $i7) || ($i2 == $i7) || ($i3 == $i7) || ($i4 == $i7) || ($i5 == $i7) || ($i6 == $i7) ){ goto LABEL7; } }

# Cluster format: $clusters{output neuron #} {input1#,input2#,input3#,input4#,input5#,input6#,input7#}

if ($clusters{$iOutputNeuron}{"$i1" .','. "$i2" .','. "$i3" .','. "$i4" .','. "$i5" .','. "$i6" .','. "$i7"} > 0){

$clustersCtr{$iOutputNeuron}{"$i1" .','. "$i2" .','. "$i3" .','. "$i4" .','. "$i5" .','. "$i6" .','. "$i7"}++; # Counter of identical clusters

}

$clusters{$iOutputNeuron}{"$i1" .','. "$i2" .','. "$i3" .','. "$i4" .','. "$i5" .','. "$i6" .','. "$i7"} = 1;# $clusters{}{} == 1: a cluster exists

$createdClusterCtr++;

if ( $iOutputNeuron == 0 && $iSimulation == 0) {$globalClusterCounterPerNeuron++;}

}

}

return;

}

sub generatePatterns{ # Generate random patterns and assign them to minicolumns

my $ctrOutputClass = 0, $ctrObj = 0, $blobCtr = 0, $blobVal = int(rand(scalar @inpPattern));

# $blobVal is a counter switching between "XXXXX" and "XXOXX" objects, it is randomly initialized to 0 or 1 for Fig 10C, always 0 for Fig 10D

for(my $iPattern = 0; $iPattern < $N_p; ) {

LABEL3:

my @currentPattern = (0) x $N_i;

my @currentPatternStart = (0) x $N_i;

for(my $i = 0; $i < $N_e; $i++ ) {

LABEL2:

my $iInp = int(rand($N_i - $minBlob + 1));

for (my $pos = $iInp - 1; $pos < $iInp + $minBlob + 1; $pos++ ){

if ($currentPattern[$pos] == 1) {goto LABEL2}; # Regenerate an object ("blob") if its within one input from another object

}

$currentPatternStart[$iInp] = 1;

for (my $pos = $iInp; $pos < $iInp + $minBlob; $pos++ ){

$currentPattern[$pos] = int(substr($inpPattern[$blobVal], $pos - $iInp, 1)); # Add new object to the pattern

}

}

foreach my $createdPatternIndexes (@patternIndexes) {

my @currentPatternIndexes = @{ getFiringInputsOrOutputsIndexes(\@currentPattern) };

if ( join(',',@$createdPatternIndexes) eq join(',',@currentPatternIndexes) ) {goto LABEL3;} # Do not create duplicate patterns

}

LABEL8:

for(my $j = 0; $j < $N_i; $j++){

if($currentPatternStart[$j] == 1){

for (my $pos = $j; $pos < $j + $minBlob; $pos++ ){

$currentPattern[$pos] = int(substr($inpPattern[$blobVal], $pos - $j, 1)); # Store a pattern

}

}

}

$patterns[$iPattern] = [ @currentPattern ]; # @patterns stores the patterns in the format (0,1,1,0,1,...)

my @tempArray = @{ getFiringInputsOrOutputsIndexes(\@currentPattern) };

$patternIndexes[$iPattern] = [ @tempArray ]; # @patternIndexes stores the indexes of the firing neurons in the format (1,2,4,..)

$correctOutputNeuron[$iPattern] = $ctrOutputClass; # @correctOutputNeuron stores the classification of patterns into minicolumn outputs

$iPattern++;

$ctrOutputClass++;

if ($ctrOutputClass == $N_o) {$ctrOutputClass = 0;}

$blobCtr++;

$blobVal++; # For Fig 10C, switch the $blobVal counter from "XXXXX" to "XXOXX" and vice versa

if($blobVal >= scalar @inpPattern){

$blobVal = 0;

}

if($corrPatterns == 1){

if ($blobCtr < scalar @inpPattern){

goto LABEL8; # For Fig 10C, create a pattern "XXXXX" for a corresponding "XXOXX" and vice versa, assign it to a different minicolumn

}else{

$blobCtr = 0;

$blobVal = int(rand(scalar @inpPattern))

}

}else{

if ($blobCtr == scalar @inpPattern){

$blobCtr = 0;

$blobVal = int(rand(scalar @inpPattern))

}

}

}

}

sub updateClusters { # Update clusters with rewards

my $reward = $_[0];

my @firingInputIndexes = @{ $_[1] };

my @firingOutputNeurons = @{ $_[2] };

if ($flagTrialFiring == 0 && $reward > 0 ){ return; }

$ctr2 = 0;

my @firingOutputIndexes = @{getFiringInputsOrOutputsIndexes (\@firingOutputNeurons) };

my @iter1 = @firingInputIndexes, @iter2 = @firingInputIndexes, @iter3 = @firingInputIndexes, @iter4 = @firingInputIndexes;

my @iter5 = @firingInputIndexes, @iter6 = @firingInputIndexes, @iter7 = @firingInputIndexes;

if( $n_c <=6 ) { @iter7 = (9999); }

if( $n_c <=5 ) { @iter6 = (9999); }

if( $n_c <=4 ) { @iter5 = (9999); }

if( $n_c <=3 ) { @iter4 = (9999); }

if( $n_c <=2 ) { @iter3 = (9999); }

if( $n_c <=1 ) { @iter2 = (9999); }

foreach my $iFiringOutputNeuron (@firingOutputIndexes){ # Loop over firing output neurons and firing input neurons

my $updatedClusters = "";

foreach my $i (keys %{$clusters{$iFiringOutputNeuron}}){

$ctr2++;

my @iArray = grep { $_ < 9999 } split(/,/, $i);

if ($clusters{$iFiringOutputNeuron}{$i} > 0 && are_all_elements_present(\@iArray, \@firingInputIndexes) == 1 ){

if ( index($updatedClusters, $i) == -1 ){ # Add reward if the cluster had not been added reward for before

$clustersWt{$iFiringOutputNeuron}{$i} += $reward;

$updatedClusters .= "$i" . '|';

}

}

}

}

return;

}

sub getFiringOutputWeights { # Get weights of output neurons given an input pattern

my @firingInputIndexes = @{ $_[0] };

my @firingOutputNeurons = (0) x ($N_o * $internalWorlds);

my @outputWeight = (0) x ($N_o * $internalWorlds);

my @iter1 = @firingInputIndexes, @iter2 = @firingInputIndexes, @iter3 = @firingInputIndexes, @iter4 = @firingInputIndexes;

my @iter5 = @firingInputIndexes, @iter6 = @firingInputIndexes, @iter7 = @firingInputIndexes;

if( $n_c <=6 ) { @iter7 = (9999); }

if( $n_c <=5 ) { @iter6 = (9999); }

if( $n_c <=4 ) { @iter5 = (9999); }

if( $n_c <=3 ) { @iter4 = (9999); }

if( $n_c <=2 ) { @iter3 = (9999); }

if( $n_c <=1 ) { @iter2 = (9999); }

foreach my $iOutputNeuron (0..$#firingOutputNeurons) { # Loop over output neurons and firing input neurons

my $updatedClusters = "";

my $iCountClusters = 0;

foreach my $i (keys %{$clusters{$iOutputNeuron}}){

my @iArray = grep { $_ < 9999 } split(/,/, $i);

if ($clusters{$iOutputNeuron}{$i} >= 1 && are_all_elements_present(\@iArray, \@firingInputIndexes) == 1 ){

if ( index($updatedClusters, $i) == -1 ){

$outputWeight[$iOutputNeuron] += ($clustersCtr{$iOutputNeuron}{$i} + 1) * $clustersWt{$iOutputNeuron}{$i};

$updatedClusters .= "$i" . '|';

}

}

}

}

return \@outputWeight;

}

sub getFiringInputsOrOutputsIndexes{ # Convert an array of the type (0, 1, 0, 1, 1, ...) into an array of the indexes of

# values that are equal to 1, of the type (1, 3, 4, ...)

my @object = @{ $_[0] };

my @firingList = ();

my $ctr = 0;

foreach my $i (@object){

if ($i == 1){

push(@firingList, $ctr);

}

$ctr++;

}

return \@firingList;

}

sub getMaxWeightNeuron { # Get the neuron with max weight

my @myarray = @{ $_[0] };

my $maxval = $myarray[0];

foreach my $i ( 0..$#myarray ){

if ($maxval < $myarray[$i]){

$maxval = $myarray[$i];

}

}

my @indexArr;

foreach my $i ( 0..$#myarray ){

if ($maxval == $myarray[$i]){

push (@indexArr, $i);

}

}

my $iPick = int(rand($#indexArr + 1)); # If more than one neuron has the same max weight pick one randomly

return $indexArr[$iPick];

}

sub layer2response { # Return output of L2/3 neurons

my $trainingPattern = $_[0];

my $layer2match = $layer2[$_[1]];

if ( index($trainingPattern, $layer2match) != -1 ) {

return 1;

}else{

return 0;

}

}

sub randNH { # Generate cluster inputs

my $N_i = $_[0];

my $H = $_[1];

my $i1 = rand($N_i + ($H-1) * $F);

if ($i1 >= $N_i) {

$i1 = $N_i + int(($i1 - $N_i) / $F)

}else{

$i1 = int($i1)

}

return $i1;

}

sub are_all_elements_present { # Check if each element of the first array exists in the second array

my ($array_ref1, $array_ref2) = @_;

my %elements_of_array2 = map { $_ => 1 } @$array_ref2;

foreach my $elem (@$array_ref1) {

return 0 unless exists $elements_of_array2{$elem};

}

return 1; # All elements were found

}
